# Supplementary figures and images for: Divergent patterns of meiotic double strand breaks and synapsis initiation dynamics suggest an evolutionary shift in the meiosis program between American and Australian marsupials
Source: Front Cell Dev Biol. 2023 Apr 25;11:1147610. doi: 10.3389/fcell.2023.1147610 (PMC10166821; doi:10.3389/fcell.2023.1147610)

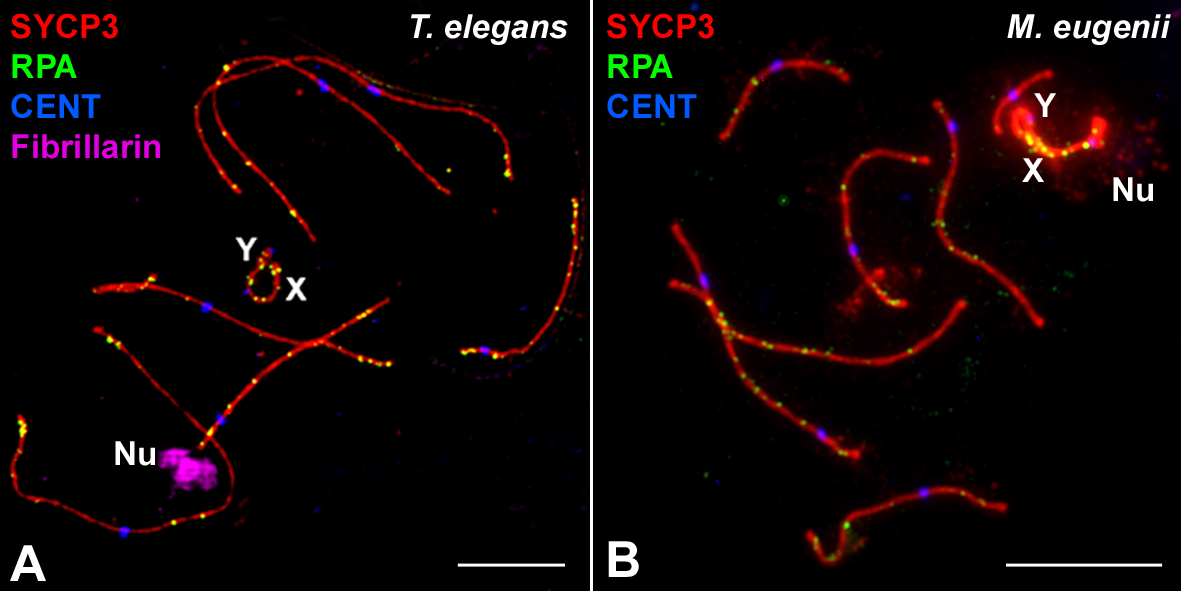

Supplement: Supplementary file 1 [file Image1.TIF]
